# The role of AGEs in skeletal muscle atrophy and the beneficial effects of exercise

**DOI:** 10.3389/fmed.2025.1626570

**Published:** 2026-01-02

**Authors:** Xinru Wu, Shuai Hu, Wei Miao, Fei Shen, Li Jiang

**Affiliations:** 1 Department of Internal Medicine, Kunshan Integrated TCM and Western Medicine Hospital, Suzhou, China; 2 Sports and Health Collaborative Innovation Center for Fitness Promotion, Jiangsu Normal University, Xuzhou, China; 3 Institute of Physical Education, Jiangsu Normal University, Xuzhou, China; 4 Department of Internal Medicine, Taicang Hospital of Traditional Chinese Medicine, Suzhou, China

**Keywords:** skeletal muscle atrophy, advanced glycation end-products, AGEs-RAGE signaling axis, aging, inflammation, oxidative stress, mitochondria, exercise

## Abstract

**Background:**

Advanced Glycation End Products (AGEs) are associated with the aging and atrophy of skeletal muscle. Their pathogenic mechanism mainly involves the binding of AGEs to their own receptors, which in turn triggers a series of pathological reactions. Exercise is considered an effective intervention method, as it can regulate the level of AGEs, thereby alleviating skeletal muscle atrophy.

**Objective:**

This study aims to review the latest research progress on skeletal muscle atrophy induced by AGEs and the beneficial effects of exercise.

**Methods:**

Relevant literature was searched from the establishment of databases (PubMed, Web of Science, Embase, and Scopus) to May 2025. The search terms were: “advanced glycation end products, receptor for advanced glycation end products, skeletal muscle, skeletal muscle atrophy, sarcopenia, aging, diabetes mellitus, obesity, exercise, aerobic training, resistance training, high-intensity interval training”. Literature was included based on the following criteria: (a) Studies focusing on the mechanism of skeletal muscle atrophy induced by AGEs and the content related to exercise regulating AGEs levels; (b) Priority was given to literature published in the past 5 years with outstanding quality, relevance, or innovation. Finally, 138 pieces of literature were included for the review.

**Results and conclusions:**

AGEs bind to the receptor for advanced glycation end products (RAGE), which leads to a decrease in muscle protein synthesis, an increase in protein degradation, impairment of muscle fiber regeneration ability, and aggravation of myocyte apoptosis, thereby inducing or exacerbating skeletal muscle atrophy. Exercise can reduce the harmful effects of AGEs on muscle mass. Specifically, exercise can reduce the formation of AGEs by improving insulin sensitivity and glucose utilization, as well as alleviating chronic inflammation and oxidative stress. Additionally, exercise enhances the metabolic capacity of the kidneys for AGEs. These findings provide new insights for the development of drug regimens targeting the “AGEs-RAGE” axis and exercise interventions. In the future, in-depth clarification of the role of AGEs in the pathogenesis of skeletal muscle atrophy and the improvement mechanism mediated by exercise will provide an important basis for the prevention and treatment of sarcopenia related to aging and metabolic disorders.

## 1 Introduction

Skeletal muscle, a principal tissue responsible for human locomotion and metabolic regulation, constitutes approximately 40% of adult body mass. Muscle atrophy diminishes muscle contractile function and mobility, exacerbating frailty-associated fall risks and increasing mortality rates. Previous estimates suggest that accelerated population aging will escalate the global burden of age-related muscle atrophy to surpass 1.4 billion cases by 2030 (1). Skeletal muscle loss can be caused by a variety of factors, including aging, metabolic disorders, lack of exercise, malnutrition, disease, and drug effects (2, 3). Emerging evidence identifies advanced glycation end-products (AGEs) as critical mediators in the pathogenesis and progression of skeletal muscle atrophy (4, 5). AGEs are pathogenic compounds generated through non-enzymatic glycation reactions between reducing sugar-derived carbonyl moieties and amino residues in biomacromolecules. Pathological accumulation of AGEs exhibits strong pathophysiological correlations with age-associated degenerative diseases, metabolic syndrome progression, and carcinogenesis (6, 7). Conversely, maintaining homeostatic AGEs concentrations represents a demonstrated intervention strategy for extending health span and attenuating senescence (8, 9). Regulatory determinants of AGEs accumulation—including chronological age, dietary patterns, glycemic status, exercise frequency, and pathological comorbidities—show significant overlap with sarcopenia risk factors (10, 11). Specifically, dermal AGEs deposition measured by autofluorescence has predictive value for sarcopenia development among community-dwelling older adults (12). Chronic AGEs exposure disrupts muscle protein homeostasis, accelerates cellular senescence, and reduces muscle mass (13). The accumulation of AGEs in skeletal muscle, caused by high-sugar and high-fat diets, can lead to skeletal muscle atrophy, the transformation of muscle fibers from fast to slow, and lipid accumulation (14). Although existing studies identify the receptor of advanced glycation endproducts (RAGE)-mediated proteostasis disruption and myofiber loss as primary mechanisms for AGEs-induced atrophy (15, 16), the potential mechanisms of AGEs-RAGE signaling axis aberrant activation inducing skeletal muscle atrophy remain unclear. Hence, this review summarizes the regulatory effects of the AGEs-RAGE signaling axis on protein synthesis and degradation, cell regeneration and apoptosis of skeletal muscle, and the impact of exercise on alleviating skeletal muscle atrophy by reducing AGEs formation and accumulation.

## 2 AGEs and their pathogenic mechanism

### 2.1 Formation and metabolism of AGEs

**Figure 1:**
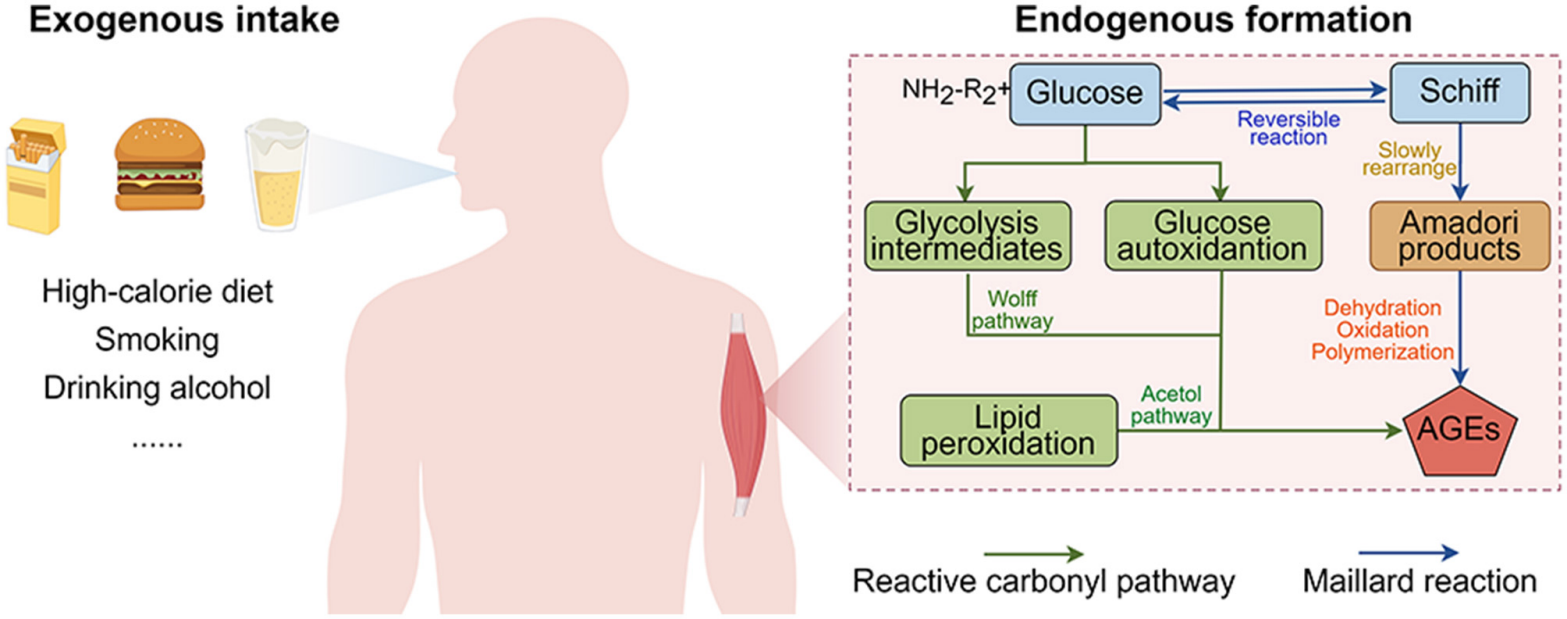
Origin of advanced glycation end-products (AGEs) in the body.

AGEs accumulate through distinct endogenous and exogenous pathways under physiological conditions (Figure 1). Endogenous AGEs predominantly derive from the Maillard reaction's terminal phase, progressing through three mechanistically distinct stages: (1) glucose-protein condensation, (2) cyclization, and (3) stereochemical rearrangement. In the initial stage of the Maillard reaction, the carbonyl group of reducing sugars undergoes reversible reactions with amino groups from proteins or amino acids to form unstable Schiff base adducts. These adducts are subsequently converted into relatively stable Amadori products (early glycation products) via cyclization and intramolecular rearrangement. Subsequently, Amadori products undergo either direct oxidative cleavage or processes of dehydration, oxidation, and rearrangement to ultimately form stable and irreversible AGEs (17). Beyond the Maillard reaction, the formation of AGEs can also occur independently through glucose autoxidation (Wolff pathway), lipid peroxidation (Acetol pathway), and direct oxidative cleavage of Schiff bases (Namiki pathway), all of which generate reactive dicarbonyl compounds (18). Under physiological conditions, AGEs accumulate slowly through both intracellular and extracellular formation. However, aging, metabolic dysregulation, hyperglycemia, and high-AGEs diets can accelerate the formation of endogenous AGEs (19, 20).

Exogenous AGEs are the primary source of bodily AGEs, predominantly found in highly processed foods such as fried items and sugar-rich products (21). Simultaneously, factors such as smoking, alcohol abuse, and exposure to environmental pollutants can increase the formation of AGEs (22, 23). After consumption, exogenous AGEs undergo gastrointestinal digestion, epithelial absorption, and systemic circulation before accumulating in organ tissues (24). Research indicates that 30%−40% of dietary AGEs are partially broken down by gastric acid and absorbed as peptides or free forms in the small intestine, while unabsorbed AGEs undergo colonic microbial metabolism before being excreted. In healthy individuals with normal renal function, approximately 30% of ingested AGEs are excreted through urine (24). Consequently, the kidneys and intestines serve as vital organs for AGEs metabolism. Modern high-calorie diets significantly increase the intake of exogenous AGEs, compounded by endogenous production pathways that elevate circulating AGE levels, collectively inducing pathological alterations in tissues and organs that drive metabolic disorders, cardiovascular diseases, neurodegenerative conditions, and accelerated aging (25, 26). Therefore, understanding the generation of AGEs is crucial for disease prevention and health maintenance.

### 2.2 The pathogenesis of AGEs

When excessive AGEs accumulate beyond the body's metabolic clearance capacity, they deposit in tissues and organs, initiating or exacerbating pathological conditions. The pathomechanisms of AGEs include: (1) direct crosslinking between reducing sugars and biomolecules (particularly extracellular matrix components), inducing structural-functional cellular alterations; (2) receptor-mediated signaling activation causing tissue damage. AGEs receptors encompass receptor complexes (AGE-R1, R2, R3) and scavenger receptors (SR-A, LOX-1, FEEL-2, CD36), with current research focusing on interactions with RAGE (27, 28).

As a member of the immunoglobulin superfamily, RAGE is ubiquitously expressed on cell surfaces. It triggers disease pathogenesis through downstream pathway activation upon engagement with AGEs: (1) It activates nuclear factor kappa-B (NF-κB) via the Ras-extracellular regulated protein kinases 1/2 (ERK1/2) and protein kinase C (PKC)/mitogen-activated protein kinase (MAPK) pathways, or directly induces proinflammatory cytokines (MCP-1, IL-6, TNF-α) and adhesion molecules (ICAM-1, VCAM-1) through janus kinase (JAK)/signal transducer and activator of transcription (STAT) signaling, promoting inflammation, proliferation, or apoptosis (29). (2) The enhancement of reactive oxygen species (ROS) production through nicotinamide adenine dinucleotide phosphate hydrogen (NADPH) oxidase and mitochondrial pathways induces oxidative stress. Excessive ROS accelerates the formation of AGEs and activates NF-κB, creating inflammatory-oxidative loops during gluco-oxidative stress (30). (3) The suppression of the phosphoinositide 3-kinase (PI3K)/protein kinase B (Akt) pathway inhibits insulin-stimulated glucose transporter 4 (GLUT4) translocation, resulting in insulin resistance and glucose dysregulation (31, 32). (4) The induction of endoplasmic reticulum (ER) stress-mediated inflammation/apoptosis occurs when AGEs accumulated in the ER disrupt protein folding and crosslinking of mitochondrial respiratory chain proteins, suppressing adenosine triphosphate (ATP) synthesis while increasing ROS, and triggering apoptotic/inflammatory signaling (33). Collectively, these pathways cause cellular dysfunction/death, ultimately leading to the pathogenesis of chronic diseases.

## 3 Molecular mechanism of skeletal muscle atrophy

Skeletal muscle atrophy primarily results from decreases in both muscle fiber quality and quantity (34). Strategies to reverse muscle wasting include promoting protein synthesis and increasing fiber number (35, 36). Protein synthesis is principally regulated by the PI3K/Akt/mammalian target of rapamycin (mTOR) pathway, whereas protein breakdown is dependent on both the ubiquitin-proteasome system (UPS) and the autophagy-lysosomal system (ALS) (37). The activation of skeletal muscle satellite cells plays a crucial role in myofiber regeneration and tissue repair (38). Insulin and IGF-1 enhance protein synthesis in skeletal muscle while stimulating the activation of muscle satellite cells. Phosphorylation of Akt, mediated by insulin-like growth factor-1 (IGF-1), increases mTORC1 activity, elevates p70 ribosomal protein S6 kinase (p70s6k) levels, and suppresses eIF4E-binding protein 1 (4E-BP1) function, collectively promotingprotein production. Through the suppression of glycogen synthase kinase-3β (GSK-3β) activity and the downregulation of eukaryotic initiation factor-2B (EIF2B) expression, Akt further enhances protein biosynthesis in skeletal muscle (39, 40). Moreover, as a central regulator of protein metabolism, Akt phosphorylates and inhibits forkhead transcription factor O subfamily member 3a (FoxO3a), downregulating UPS-associated genes Atrogin-1 and MuRF1 to reduce protein degradation and muscle atrophy (41). Myostatin acts as a limiting factor for skeletal muscle growth, where the myostatin-Smad2/3 signaling pathway accelerates muscle protein degradation through activation of the UPS pathway (42). In addition, reduced ALS activity leads to the accumulation of macromolecular proteins and damaged organelles. Dysfunctional mitochondria-generated ROS directly activate UPS to promote protein degradation and muscle loss. In contrast, the accumulation of ROS also induces the release of cytochrome C (Cyt-C) and activates the apoptotic pathway through caspase cascades (43). Chronic inflammation is another critical factor in muscle atrophy, where proinflammatory TNF-α activates the NF-κB pathway, resulting in skeletal muscle proteolysis and myocyte apoptosis (44, 45). Thus, muscle atrophy arises from the complex interplay of various mechanisms, resulting in diminished muscle functionality, metabolic dysregulation, and decreased myofiber quantity and size. These pathological changes can be mitigated by physical exercise through multiple mechanisms: attenuating inflammation, boosting anabolic processes, enhancing autophagic clearance, inhibiting myostatin signaling and apoptosis, and activating muscle progenitor cells (46–48).

## 4 The regulatory role of AGEs in skeletal muscle atrophy

**Figure 2:**
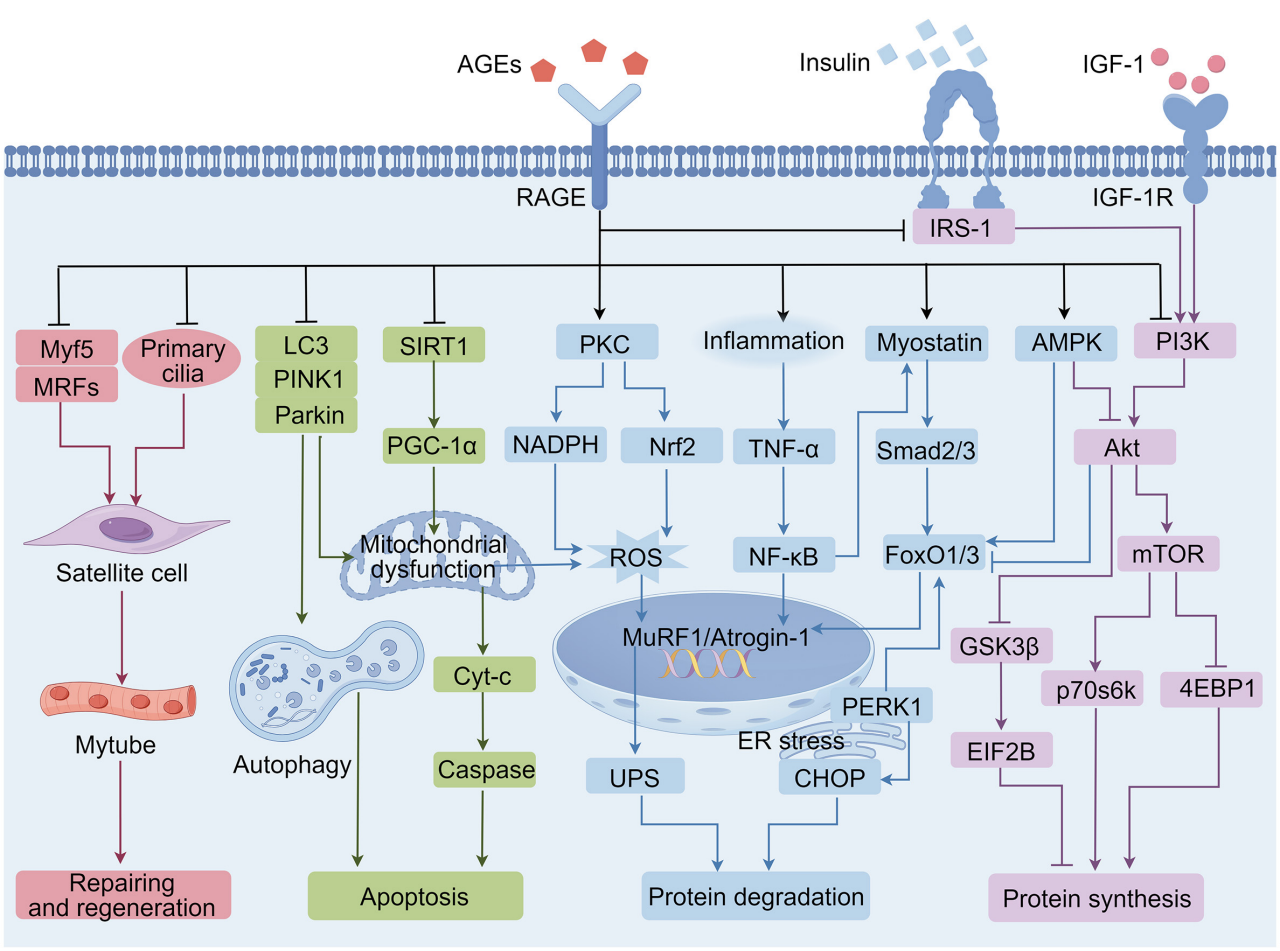
Underlying mechanisms of advanced glycation end-products (AGEs) leading to skeletal muscle atrophy. Akt, serine/threonine kinase; AMPK, adenosine 5'-monophosphate; Atrogin-1, muscle atrophy f-box; Cyt- C, Cytochrome C; CHOP, C/EBP-homologous protein; 4E-BP1, eIF4E-binding protein 1; EIF2B, eukaryotic initiation factor-2B; ER, endoplasmic reticulum; FoxO1/3, forkhead transcription factor O subfamily member 1/3; GSK-3β, glycogen synthase kinase-3β; IGF-1, insulin-like growth factor-1; IGFR, insulin-like growth factor receptor; IRS-1, insulin receptor substrate 1; LC3, microtubule-associated protein 1 light chain 3; MRFs, myogenic regulatory factors; mTOR, mammalian target of rapamycin; MuRF1, muscle RING-finger protein-1; Myf5, myogenic factor 5; NADPH, nicotinamide adenine dinucleotide phosphate hydrogen; NF-κB, nuclear factor kappa beta; Nrf2, nuclear factor erythroid 2-related factor 2; Parkin, RBR E3 ubiquitin protein ligase; PERK1, protein kinase RNA–like endoplasmic reticulum kinase 1; PINK1, PTEN induced putative kinase 1; PI3K, phosphatidylinositol 3-kinase; PGC-1α, peroxisome proliferator-activated receptor-γ coactivator-1α; PKC, protein kinase C; p70s6k, p70 ribosomal protein S6 kinase; RAGE, receptor for AGEs; ROS, reactive oxygen species; Smad2/3, mothers against decapentaplegic 2/3; SIRT1, sirtuin1; TNF-α, Tumor necrosis factor-α; UPS, ubiquitin-proteasome system.

Dysregulated RAGE expression and activation may serve as a biomarker for muscular atrophy (16). Elevated levels of AGEs lead to the upregulation of RAGE in skeletal muscles (49). This synergistic interaction coordinates downstream signaling cascades, contributing to skeletal muscle wasting through interference with protein homeostasis, inhibition of satellite cell activation, and induction of apoptotic pathways (Figure 2).

### 4.1 AGEs inhibit protein synthesis in skeletal muscle

The PI3K/Akt/mTOR signaling pathway primarily regulates protein synthesis in skeletal muscle. Impaired PI3K/Akt/mTOR signaling in skeletal muscle suppresses protein synthesis, ultimately leading to muscle atrophy (50). Elevated AGEs are closely associated with this process, particularly in aged individuals and diabetic patients. Treatment with AGEs in C2C12 myoblasts downregulates nearly all phosphorylation sites associated with PI3K/Akt/mTOR signal transduction (51). Furthermore, a 16-week high-AGEs diet in rodents leads to a reduction in skeletal muscle mass, a decrease in muscular strength, and a downregulation of phosphorylation of Akt at Ser473 and p70s6k at Thr389 within protein synthesis pathways (52). However, AGEs-RAGE-induced Akt downregulation and skeletal muscle atrophy could be drastically reversed by treatment with IGF-1 (49). This indicates that AGEs-mediated suppression of the PI3K/Akt/mTOR signaling pathway potentially drives age-associated muscle atrophy through impaired protein synthesis. As a critical nutrient/energy sensor, mTOR activation via AGEs-RAGE signaling suppresses protein synthesis by reducing the phosphorylation of mTOR, p70s6k, and 4E-BP1, ultimately causing skeletal muscle atrophy in mice (53). Notably, this study also revealed that AGEs induce ER stress. The ER-transmembrane kinase protein kinase RNA-like endoplasmic reticulum kinase (PERK) inactivates the EIF2α to decrease protein synthesis while activating C/EBP homologous protein (CHOP) to accelerate protein degradation (13, 53).

Skeletal muscle tissue acts as one of the primary target organs for insulin. The binding of insulin and IGF-1 to sarcolemmal receptors triggers the insulin receptor substrate 1 (IRS-1)/PI3K/Akt signaling pathway and phosphorylation of mTOR pathway-related sites, thereby facilitating anabolism and glucose uptake (54). However, various factors—especially chronic high-fat/high-sugar (HFD) diets, physical inactivity, and obesity—result in decreased insulin sensitivity in target organs, leading to insulin resistance, reactive hyperglycemia, and hyperinsulinemia (55–57). Chronic hyperglycemia accelerates the formation of endogenous AGEs, which further exacerbates insulin resistance and negatively impacts skeletal muscle protein synthesis (58). For example, AGEs may impair insulin signaling in skeletal muscle by forming RAGE/IRS-1/steroid receptor coactivator (Src)/PKCα complexes both *in vitro* and *in vivo* (59). AGEs inhibitors can restore insulin sensitivity and partially mitigate the insulin resistance caused by AGEs-mediated downregulation of insulin signaling transduction in skeletal muscle (60). Therefore, AGEs-RAGE signal axis likely suppress protein synthesis by modulating multiple signaling pathways. Lowering endogenous AGEs may serve as a promising strategy to enhance protein synthesis, preventing and alleviating muscle atrophy, particularly in diabetes- and aging-associated muscle atrophy.

### 4.2 AGEs accelerate the protein degradation in skeletal muscle

#### 4.2.1 AGEs activate ubiquitin-proteasome system in skeletal muscle

UPS is mainly responsible for the protein degradation in skeletal muscle. The transcriptional upregulation of atrophy-related genes, such as MuRF1 or atrogin-1, activates the UPS, accelerating proteolysis and inducing skeletal muscle atrophy. Previous research has shown that adenosine 5'-monophosphate (AMPK)-dependent modulation of AGEs-RAGE in diabetic mice upregulates atrogin-1 expression, leading to skeletal muscle protein degradation through UPS pathways (49). Chronic inflammation triggers skeletal muscle protein degradation and is a key pathogenic mechanism behind muscle atrophy. The interaction of AGEs-RAGE activates NF-κB signaling, which polarizes macrophages toward proinflammatory phenotypes and triggers the release of TNF-α and IL-6 (61, 62). RAGE blockade partially reverses these effects, indicating that AGEs/RAGE axis activation induces systemic chronic low-grade inflammation (63, 64). This induces the expression of MuRF1/Atrogin-1 through the NF-κB-dependent pathway and the abrogation of Akt-mediated phosphorylation inhibition on FoxO3a, ultimately leading to protein degradation and skeletal muscle atrophy. Notably, several inflammation-related metabolites (such as sarcosine, tryptophan, and arginine) exert an inhibitory effect on the formation of AGEs. More importantly, alterations in these metabolites under aging or chronic disease conditions can exacerbate systemic inflammation (65, 66). For example, plasma sarcosine levels are significantly decreased in elderly patients with sarcopenia. Sarcosine not only promotes muscle regeneration by inducing the anti-inflammatory polarization of macrophages (66), but also is negatively correlated with AGEs in plasma (67). This suggests that the dysregulation of metabolites in the body may be a crucial factor that accelerates AGEs formation and thereby leads to muscle atrophy. Further investigation into the underlying mechanisms is of great significance for understanding the metabolic dimension behind AGE-induced atrophy.

High levels of oxidative stress significantly contribute to protein degradation-mediated muscle atrophy, predominantly caused by the overproduction of ROS. When ROS levels in muscle cells exceed the capacity of endogenous antioxidant systems during aging or metabolic stress, significant oxidative cellular injury occurs. AGEs stimulate ROS generation in muscle cells, exacerbating oxidative stress in skeletal musculature. The activation of RAGE in healthy mice elevates muscular ROS levels and induces distinct muscle wasting characteristics, manifested as reductions in muscle mass and cross-sectional area (68). The AGEs-RAGE axis induces ROS production in myocytes by facilitating the phosphorylation of PKCα and the regulatory subunit p47phox of NADPH oxidase (NOX2) (69). Additionally, AGEs suppress the expression of antioxidant enzymes superoxide dismutase 2 (SOD2) and peroxiredoxin III (PRXIII) in skeletal muscle (70), intensifying oxidative stress, primarily via the downregulation of nuclear factor erythroid 2-related factor 2 (Nrf2)/heme oxygenase 1 (HO-1) signaling (71). Furthermore, the oxidative microenvironment created by AGEs potentially involves disturbed sirtuin1 (SIRT1) activity. As a NAD+-sensitive deacetylase, SIRT1 serves crucial protective functions against oxidative damage through epigenetic regulation. Prolonged AGE exposure reduces AGER1 and SIRT1 expression in rodent skeletal muscle, elevating systemic oxidative stress while promoting metabolic dysfunction-associated insulin resistance (72). Beyond ROS suppression, SIRT1 mitigates muscle atrophy by inhibiting FoxO1/3 activation-mediated upregulation of atrogin-1 and MuRF1 (73). Elevated ROS directly activates UPS, accelerating skeletal muscle proteolysis and mass reduction (74). Concurrently, AGEs-derived ROS activates PERK/FoxO1 signaling to upregulate Atrogin-1 expression, culminating in murine skeletal muscle atrophy (13). Conversely, AGEs toxicity inhibition reduces RAGE expression, diminishes lipid oxides levels in skeletal muscle, and downregulates MuRF1 transcription/translation, consequently alleviating age-related muscle atrophy (75).

#### 4.2.2 . AGEs reduce the autophagy-lysosomal system of skeletal muscle

ALS serves as a vital mechanism for clearing intracellular abnormal macromolecular proteins and organelles, thereby maintaining skeletal muscle mass homeostasis under various stress conditions. Animal studies have demonstrated that the muscle-specific knockout of autophagy-related protein 7 or the colchicine-induced blockade of autophagic flux results in the massive accumulation of abnormal protein aggregates in skeletal muscle, impairing muscle fiber structure and quality (76, 77). Similarly, reduced autophagic activity in skeletal muscle has been observed in aged individuals and diabetic patients, showing a strong correlation with the pathogenesis of muscle atrophy (78, 79). This suggests that maintaining normal autophagic activity is essential for ensuring regular protein metabolism in skeletal muscle. Research indicates that AGEs may not enhance protein degradation via ALS but rather impair its activity, potentially triggering apoptosis and ultimately leading to muscle atrophy (80). For example, *in vitro* treatment of murine skeletal muscle with AGEs for 6 h significantly reduced the expression of microtubule-associated protein 1 light chain 3 (LC3), a marker of autophagosome formation (53). Both AGEs-induced diabetic mice and D-galactose-treated aging models exhibited impaired autophagy in skeletal muscle, characterized by a decreased LC3B-II/I ratio and elevated sequestosome 1 (P62) expression. D-pinitol administration ameliorated diabetes- and age-related muscle atrophy by reversing autophagic dysfunction (80). In conclusion, AGEs disrupt both the UPS and ALS, while reducing endogenous AGEs levels helps preserve the normal function of these proteolytic systems.

### 4.3 AGEs damage cell regeneration and repair of skeletal muscle

In adult skeletal muscle, satellite cells (MuSCs) constitute about 1% of the total skeletal muscle cells and are typically quiescent. However, the formation of skeletal muscle and post-injury regeneration are critically dependent on their presence (81). The myogenic process involves the activation of resting satellite cells, their subsequent proliferation and differentiation into precursor myocytes, cellular fusion to form multinucleated myotubes, and the final development into functional muscle fibers (82). Upon sustaining damage, dormant satellite cells are activated through asymmetric cell division, which triggers the expansion and specialization of these muscle-resident stem cells (83). The paired-box transcription factor paired box 7 (Pax7) specifically upregulates genes that drive satellite cell expansion while concurrently repressing genetic programs that initiate muscle lineage commitment (84). Myogenin (MyoG) collaborates with Mrf4 to initiate terminal differentiation, transforming flattened cells into spindle-shaped forms that fuse into myotubes and mature into muscle fibers (85).

RAGE exhibits a transient expression in muscle satellite cells subsequent to skeletal muscle injury and plays a crucial role throughout the entire myogenic differentiation process. Following acute skeletal muscle damage, the released S100B protein binds to the RAGE receptor, triggering proliferation mediated by ERK1/2 and activation of myogenic pathways through p38MAPK (86). During terminal differentiation stages, the activation of RAGE signaling enhances muscle regeneration by suppressing Pax7 expression via myogenin (87). However, sustained high levels of AGEs under pathological conditions significantly impair the proliferation, differentiation, and fusion of skeletal muscle satellite cells. Treatment with AGEs significantly increases apoptotic rates in C2C12 myoblasts, an effect that is counteracted by inhibition of AGEs through aminoguanidine (88). A chronic high-AGEs diet in aged mice showed unaffected Pax7 gene expression but significant downregulation of myogenic differentiation (MyoD) and myogenic factor 5 (Myf5) transcription in skeletal muscle, potentially leading to impaired myoblast proliferation/fusion, hindered myofiber formation, and accelerated muscle mass loss (52). Similarly, the subcutaneous injection of DNA aptamers targeting AGEs (which can inhibit the toxicity of AGEs) in SAMP8 accelerated aging mice, while reducing RAGE expression and improving the phenotype of muscle atrophy, did not alter the declining trend of skeletal muscle Pax7 (75). The research indicates that the direct inhibitory effect of the AGEs-RAGE signaling pathway on muscle regeneration is associated with the downregulation of MRFs.

Beyond the direct modulation of MRFs, primary cilia act as crucial regulators that promote satellite cell activation and orchestrate skeletal muscle differentiation and regeneration. The activation of the AGEs-RAGE pathway impairs ciliogenesis in myoblasts, whereas inhibition of RAGE eliminates AGEs-induced ciliary damage, enhancing myogenic differentiation/repair and increasing myotube diameter (89). Primary cilia transmit multiple signaling pathways, including the Sonic hedgehog (SHH), Wnt signaling pathway, and Notch signaling pathway (90, 91). Dysregulation of these pathways suppresses myogenic regulatory factors (MyoD, MyoG, Myf4, Myf5), impairing myoblast differentiation in muscle fibers and downregulating myogenic regulators, ultimately causing skeletal muscle loss (92). Therefore, defective ciliogenesis may mediate the negative impact of AGEs-RAGE signaling on myogenesis. During aging, upregulated JAK/STAT and downregulated ERK pathways contribute to a reduced satellite cell pool, disordered regeneration cycles, and failed satellite cell activation. AGEs exposure increases STAT3 phosphorylation while suppressing ERK activation in C2C12 myoblasts, effectively blocking myogenic fusion processes (80, 93).

In the pathological state, AGEs can directly inhibit the activation of myogenic regulators, and damage the formation of primary cilia and the conduction of JAK/STAT and ERK signaling pathways, to damage the growth and repair of skeletal muscle. Although the role of skeletal muscle proliferation and regenerative decline in mediating AGEs in promoting amyotrophy has attracted more and more attention, further exploration of its systemic mechanism will provide a new perspective for the prevention and treatment of aging and metabolic disorder amyotrophy.

### 4.4 AGEs increase mitochondria-mediated apoptosis of cells in skeletal muscle

Mitochondria, as essential organelles in skeletal muscle, not only play a crucial role in energy metabolism but also act as the central link in cellular survival. The normal functioning of mitochondria is vital to maintain the quality and function of skeletal muscle. Reducing the number of mitochondria due to AGEs, the accumulation of abnormal mitochondria, and a decrease in the efficiency of mitochondrial oxidative metabolism can lead to increased myocyte apoptosis and impaired exercise capacity (94, 95). Numerous studies have reported that abnormal changes in the mitochondrial proteome occur in aging and metabolic diseases such as diabetes and obesity, with AGEs being a key limiting factor (96). Furthermore, in other organ tissues, including cardiac muscle, the damaging effects of AGEs-RAGE signaling on mitochondrial dynamics—including fission, fusion, and mitophagy—have been observed (97).

AGEs are closely associated with mitochondrial quality control and metabolic dysfunction. In chronic kidney disease (CKD) mice with muscle wasting, the gastrocnemius muscles exhibited elevated AGEs expression, accompanied by reduced PGC1-α levels and succinate dehydrogenase (SDH) activity (98). PGC1-α is a key molecular stimulator of mitochondrial biogenesis, essential for maintaining skeletal muscle mass and function. AGEs decrease mtDNA content by suppressing PGC-1α activity, ultimately causing skeletal muscle atrophy (71). In AGEs-induced diabetic atrophy models, gastrocnemius muscles displayed diminished mitochondrial membrane potential, aberrant morphology, accumulation of damaged mitochondria, and a marked reduction in mitophagy biomarkers PTEN induced putative kinase 1 (PINK1)/RBR E3 ubiquitin protein ligase (Parkin) (80). These findings suggest that AGEs impair skeletal muscle mitochondrial quality partly by suppressing the PGC-1α and PINK1/Parkin pathways. AGEs-RAGE axis impairs mitochondrial energy metabolism by reducing Complex I/III and uncoupling protein 3 (UCP3) expression, weakening respiratory capacity and fatty acid oxidation, ultimately decreasing myosin heavy chain content in skeletal muscle (94). RAGE knockout rescues AGEs-induced deficits in soleus muscle respiratory complex activities and enhances peak aerobic capacity in mice (95). Furthermore, AGEs-induced mitochondrial dysfunction correlates with diminished SIRT1 signaling. SIRT1 regulates myocyte mitochondrial biogenesis/function, and its downregulation accelerates skeletal muscle aging (99). Mastrocola et al. demonstrated that AGEs downregulate SIRT1 activity in C57Bl/6J mouse gastrocnemius, reducing mitochondrial membrane potential and creatine kinase levels (100).

Mitochondrial dysfunction appears to be a pivotal mechanism in AGEs-mediated apoptosis of muscle cells. The intrinsic mitochondrial apoptotic pathway, centered on the mitochondrial permeability transition, progresses through three key stages: mitochondrial impairment triggers cytochrome C release, which subsequently activates Caspase9 and ultimately Caspase3. Following AGEs-induced mitochondrial damage in skeletal muscle, cytochrome C released into the cytoplasm triggers apoptosome formation and Caspase9 activation, initiating a cascade reaction that activates downstream caspases and initiates apoptosis. The B-cell lymphoma-2 (Bcl-2)/BCL-2-associated X protein (Bax) ratio constitutes a critical regulatory pathway for inhibiting mitochondrial-mediated apoptosis. Studies reveal that methylglyoxal (an AGEs precursor) compromises mitochondrial function through impaired ATP synthesis and membrane potential dissipation, while upregulating Bax and downregulating Bcl-2 expression, thereby promoting cytochrome C efflux and caspase cascade activation that leads to excessive apoptosis and myotube atrophy (71).

## 5 Exercise improves AGEs accumulation and skeletal muscle mass

Currently, regular exercise is an effective intervention to reverse skeletal muscle atrophy and plays an active role in reducing the accumulation of AGEs in the body. The positive effects of exercise include reducing the endogenous formation of AGEs and limiting the accumulation following exogenous ingestion. By moderating the formation and accumulation of AGEs through exercise, one may significantly improve the quality control of skeletal muscle and alleviate aging-related muscle atrophy.

### 5.1 Exercise regulates AGEs and skeletal muscle mass

Exercise is an effective strategy for reducing the formation and accumulation of AGEs in aging individuals and patients with chronic diseases. A 6-week regimen of aerobic exercise has been shown to decrease serum AGEs concentrations in D-galactose-induced aging rat models (101). Initiating intermittent aerobic exercise during late middle age significantly reduces age-related AGEs accumulation (102). Compared to sedentary controls, individuals who engage in lifelong aerobic exercise exhibit lower circulating levels of reactive dicarbonyl metabolites-key precursors of AGEs-thereby decreasing AGEs formation (103). Additionally, middle-aged and elderly individuals who practice Tai Chi at least twice a week demonstrate reduced serum AGEs levels (104). Emerging evidence suggests that the benefits of exercise in reducing AGEs extend beyond aging to the clinical management of metabolic syndrome-associated AGEs dysregulation. Long-term aerobic exercise in overweight/obese individuals leads to improvements in body weight, BMI, and lipid profiles, and significantly lowers serum AGEs markers (N-ε-carboxymethyllysine and methylglyoxal) compared to sedentary controls (105). Resistance exercise protocols reduce AGEs precursors and enhance muscular function in diabetic populations (106). Meanwhile, high-intensity interval training (HIIT), which combines brief vigorous exercise with active recovery, improves systemic metabolic health and decreases AGEs accumulation in the elderly (107), and type 2 diabetes mellitus (T2DM) patients (108). Thus, aerobic, resistance and high-intensity interval training collectively mitigate the accumulation of AGEs associated with aging and chronic diseases.

AGEs are a crucial pathogenic factor in muscle atrophy, while resistance exercise is the preferred intervention for enhancing skeletal muscle mass. A 12-week progressive resistance training study in T2DM patients with sarcopenia demonstrated significant improvements in Hemoglobin A1c (HbA1c), five-times-sit-to-stand test scores, skeletal muscle mass, and calf circumference (109). Chronic resistance exercise yields superior benefits over aerobic training for modulating AGEs and improving muscle quality in diabetic sarcopenia patients (110). Comparative analysis reveals that long-term (9-month) resistance training surpasses aerobic exercise in reducing AGEs, decreasing body fat percentage, and increasing lean mass—with a significant inverse correlation between AGEs reduction and lean mass gain. These findings suggest that exercise may counteract AGEs-mediated skeletal muscle deterioration, with resistance training being a particularly attractive regimen for T2DM patients. Notably, a home-based resistance training study showed that a 32-week intervention maintained skeletal muscle mass and function but failed to reduce HbA1c in T2DM patients (111). These discrepancies may partially stem from variations in intervention duration (3 vs. 12 months), exercise intensity (mild vs. moderate), or study populations (asymptomatic vs. overweight/obese).

### 5.2 Pathways to reduce AGE accumulation through exercise

The precise mechanisms through which regular exercise diminishes the formation and accumulation of AGEs in the body are not fully understood. Current research indicates that exercise slows down the production and buildup of AGEs in the body partly by: (1) boosting soluble RAGE; (2) improving glycemic control; (3) combating oxidative stress; (4) alleviating chronic inflammation; and (5) enhancing renal excretion of AGEs.

#### 5.2.1 Boosting soluble RAGE

Besides full-length RAGE, there exists a C-terminal truncated variant that lacks the transmembrane domain. This variant is secreted into plasma as soluble RAGE (sRAGE or esRAGE). sRAGE binds ligands such as AGEs, preventing their interaction with membrane-bound RAGE, thereby inhibiting downstream pro-inflammatory and oxidative pathways while facilitating ligand clearance (112). Consequently, sRAGE exhibits tissue-protective properties through negative regulation of AGEs-RAGE axis-dependent pathogenic mechanisms (113, 114). Research demonstrates that increased sRAGE levels accelerate muscle regeneration and repair in aged mice following injury (115). Obese and diabetic populations exhibit significantly reduced serum sRAGE concentrations correlating with impaired glucose homeostasis (116). Exercise effectively elevates circulating sRAGE levels, enhancing sRAGE-AGEs binding to boost AGEs clearance and reduce bioavailability (117). An 8-month aerobic regimen induced a 9–22% serum sRAGE elevation, with the greatest increases observed in low-fitness individuals alongside reduced IL-6 and hypersensitive C-reactive protein (hsCRP), suggesting that exercise-induced sRAGE enhancement counteracts AGEs-mediated inflammation, particularly benefiting metabolically compromised subjects (118). Furthermore, sRAGE's interference with AGEs-RAGE signaling may involve antioxidant regulation. High-intensity training increases sRAGE while decreasing 8-isoprostane-F2α (oxidative stress biomarker) (119).

#### 5.2.2 Improving glycemic control

Hyperglycemia directly induces the formation and accumulation of AGEs through persistent non-enzymatic glycation between proteins and glucose. Circulating levels of AGEs strongly correlate with blood glucose concentrations, explaining the elevated levels of AGEs in diabetic individuals (120). Exercise-mediated improvement in glycemic control contributes to decelerating the formation and deposition of AGEs. Moderate exercise enhances glycemic control primarily by increasing skeletal muscle glucose uptake and utilization (121). The principal role of insulin involves facilitating cellular glucose uptake and utilization to maintain systemic glucose equilibrium. Insulin binds to receptors on the muscle cell membrane, triggering the IRS-1/PI3K/Akt signaling pathway to regulate GLUT4 expression and membrane translocation, thereby enhancing glucose uptake and reducing blood glucose. Under conditions of insulin resistance, impaired muscle insulin signaling disrupts skeletal muscle glucose uptake and metabolism, leading to hyperglycemia. Exercise improves glycemic control by enhancing skeletal muscle insulin signaling, upregulating sarcolemmal GLUT4, and increasing insulin sensitivity and glucose uptake (122). Additionally, exercise stimulates insulin-independent glucose uptake through AMP/ATP ratio-mediated AMPKα Thr172 phosphorylation, driving GLUT4 translocation to enhance glucose uptake and glycogenolysis for glycemic regulation (123, 124).

#### 5.2.3 Combating oxidative stress

Oxidative stress plays a central role in promoting the formation and accumulation of AGEs, as reactive oxygen species (ROS) can initiate glycation processes through interactions with sugars and proteins (125). Mitochondria serve as the primary cellular source of ROS. During oxidative phosphorylation, electron leakage from the electron transport chain (ETC) combines with oxygen to generate ROS (126). Under physiological conditions, mitochondrial and cellular antioxidant systems scavenge excess ROS through enzymatic neutralization, maintaining redox homeostasis. However, aging and chronic diseases disrupt this balance by increasing ROS production and compromising antioxidant capacity, thereby elevating oxidative stress (127). Studies demonstrate that regular exercise reduces oxidative stress by improving mitochondrial efficiency (reducing ROS generation) and upregulating endogenous antioxidants (such as SOD, TrxR, GPx, CAT) to neutralize ROS, thereby inhibiting AGEs formation (117).

#### 5.2.4 Alleviating chronic inflammation

Inflammation is a key driver that accelerates the formation of AGEs. Chronic inflammatory responses, which occur under conditions of aging, obesity, and diabetes, enhance inflammation by increasing the production of pro-inflammatory cytokines (TNF-α, IL-6, IL-1β) and activating NF-κB (128). During glycation-related oxidative stress, NF-κB further amplifies pro-inflammatory cytokine production, creating a cyclical inflammatory loop that exacerbates AGEs formation and accumulation. Additionally, inflammatory responses activate immune cells, including macrophages and dendritic cells, shifting their metabolic pathways toward glycolysis. Glycolytic metabolism generates AGEs precursors, such as methylglyoxal and glyoxal (129). Regular exercise has been proven to enhance the body's pro-inflammatory environment, promote immune cell polarization toward anti-inflammatory phenotypes, and suppress NF-κB signaling activation while reducing inflammatory biomarkers like C-reactive protein and pro-inflammatory cytokine levels (130). Moreover, exercise facilitates the expression of anti-inflammatory factors, such as muscle-derived IL-10, irisin, and meteorin-like protein (METRNL), which play crucial regulatory roles in counteracting chronic inflammation (131, 132). Through these anti-inflammatory mechanisms, exercise can mitigate AGEs formation and associated tissue damage.

#### 5.2.5 Enhancing the renal excretion of AGEs

The kidneys are critical organs for metabolizing AGEs. Studies demonstrate that proximal renal tubular epithelial cells play essential roles in clearing plasma AGEs. Approximately one-third of ingested dietary AGEs are absorbed into circulation through the gastrointestinal tract, with the remaining AGEs (about 30%) excreted via urine (133). Similarly, studies confirm marked increases in plasma AGE levels across uremic populations, regardless of diabetic status (134). Experimental models of doxorubicin-induced chronic kidney dysfunction revealed pronounced pentosidine deposition within renal tissues (135). Enhanced renal function therefore accelerates AGEs elimination and reduces systemic AGE accumulation. Exercise enhances renal capacity for AGEs clearance (136). Meta-analyses indicate that aerobic combined with resistance exercise significantly improves glomerular filtration rates and renal function in adults with chronic kidney disease (137). A 10-week aerobic interval regimen attenuated glomerulosclerosis and tubular fibrosis in diabetic-obese nephropathy mice, concurrently suppressing tissue and circulating AGEs levels (136).

**Figure 3:**
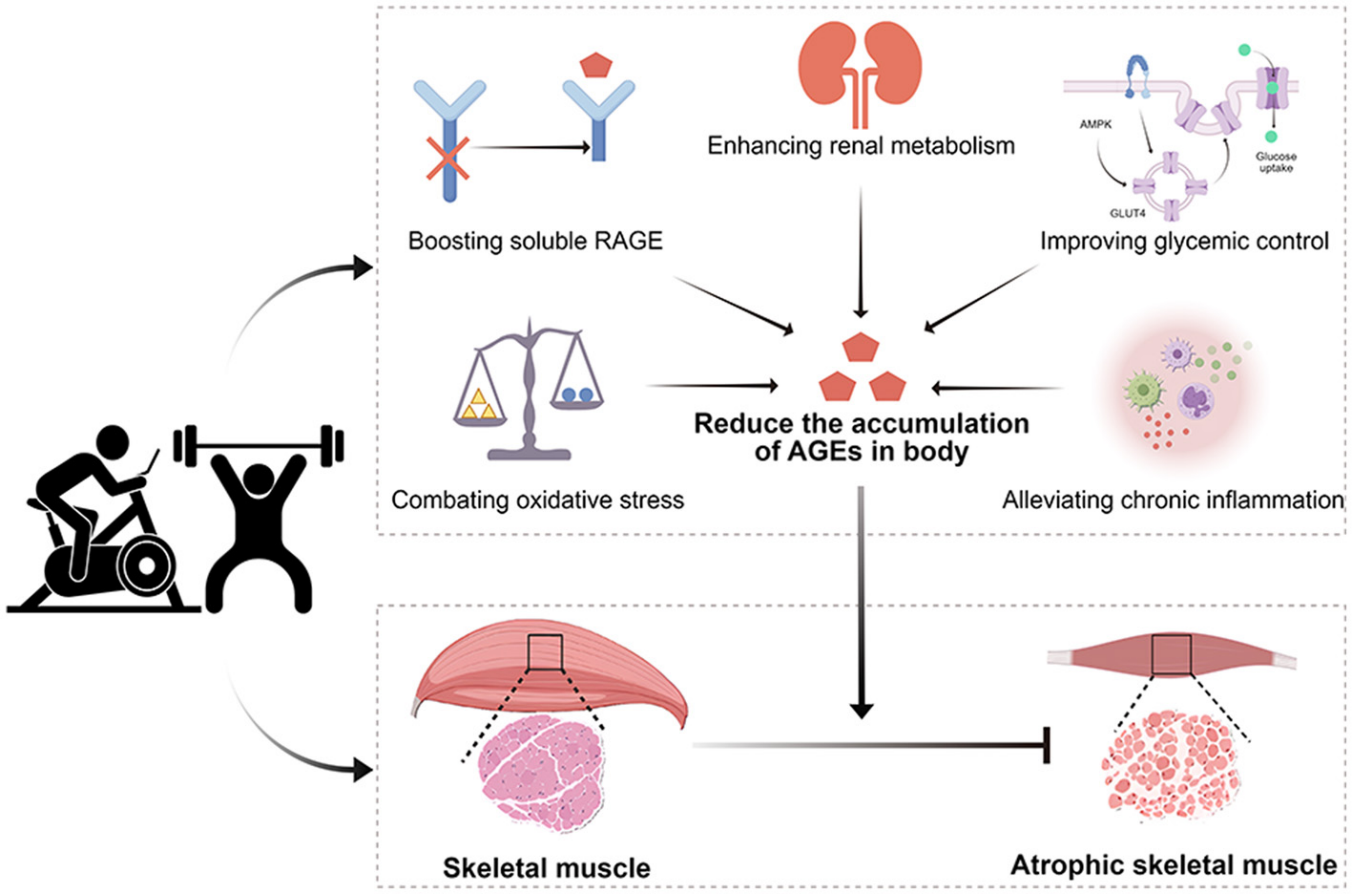
Beneficial effects of exercise on advanced glycation end-products (AGEs) and skeletal muscle atrophy.

In conclusion, exercise effectively reduces the formation and accumulation of AGEs, bringing them back to normal levels (Figure 3). Elevated levels of AGEs can lead to the deterioration of skeletal muscle structure and function. Regular exercise, combined with strategies to lower AGEs, can better improve the quality and function of skeletal muscle, particularly for the elderly and individuals with chronic diseases.

## 6 Limitations

Our study also has several limitations. First, in order to comprehensively sort out, interpret, and describe the existing research in the thematic field of “the role of AGEs in skeletal muscle atrophy and the beneficial effects of exercise”, we conducted a narrative review analysis. Although a rigorous literature search and screening strategy was adopted, meta-analysis and quantitative evaluation were not included, which may affect the objectivity of the integrated analysis. Second, the content of this review mainly focuses on basic research experiments, and most mechanism studies are carried out based on cell experiments or animal models. However, there are significant differences between mice and humans in terms of skeletal muscle fiber types and metabolic characteristics; in addition, the induction method of AGEs in animal models is inconsistent with the slow accumulation process of AGEs in humans under natural aging or metabolic disease states. In the future, more human randomized controlled studies can be conducted to deeply explore the mechanism by which AGEs induce skeletal muscle atrophy and improve the certainty of translating mechanistic conclusions into human clinical scenarios. Third, this article focuses on the AGEs-RAGE signaling axis, and clarifies the process by which it induces muscle atrophy through multiple pathways, but does not discuss other receptors of AGEs or glycosylation cross-linking. The emergence of this limitation may be related to the fact that the AGEs-RAGE signaling axis is still the main research focus in this field at present. In the future, the mechanism of action of non-RAGE pathways in AGEs-induced muscle atrophy can be further explored to fully reveal the pathogenic mechanism of AGEs. Fourth, this article mentions that some exercise modes can reduce AGEs accumulation and improve muscle atrophy, but does not further explore the intervention effects of different exercise parameters (such as the load/frequency of resistance exercise, the proportion of high-intensity periods in HIIT), the effect of exercise duration, etc.). As mentioned earlier, more human randomized controlled trials and meta-analyses are still needed to clarify the role of AGEs in inducing skeletal muscle atrophy and the beneficial effects of exercise.

## 7 Remarks and future perspectives

The accumulation of AGEs may initiate and drive the occurrence and progression of skeletal muscle atrophy. By binding to the RAGE, AGEs inhibit the insulin/IGF-1 signaling pathway, autophagic processes, satellite cell proliferation/differentiation, and mitochondrial function, while activating inflammation/oxidative stress-related pathways, the UPS, and apoptotic pathways. This series of effects leads to reduced protein synthesis, increased protein degradation, impaired myofiber regeneration, and enhanced myocyte apoptosis, ultimately triggering or exacerbating skeletal muscle atrophy. Notably, regular exercise is an effective intervention for preventing and reversing skeletal muscle atrophy. Particularly in age-related atrophy and atrophy associated with metabolic disorders, exercise can mitigate the detrimental effects of AGEs on muscle mass. Meanwhile, exercise improves insulin sensitivity and glucose utilization, thereby reducing the available substrates for glycation reactions. Additionally, the anti-inflammatory and antioxidant effects of exercise can slow down AGEs formation, and exercise can also enhance the metabolism of AGEs in the kidneys. In-depth clarification of the role of AGEs in the pathogenesis of skeletal muscle atrophy and the mechanisms underlying exercise-mediated improvement will contribute to the prevention and treatment of sarcopenia.

Since exercise may alleviate the harmful effects of AGEs on skeletal muscle, corresponding exercise therapies can be adopted to prevent and slow the progression of skeletal muscle atrophy. For high-risk populations (such as the elderly or patients with type 2 diabetes mellitus), personalized exercise programs—including resistance training (proven to be more effective in improving glycemic control and muscle mass in diabetic sarcopenia) or high-intensity interval training (effective in AGEs clearance in the elderly)—can be incorporated into clinical practice as first-line non-pharmacological intervention strategies. Meanwhile, restricting the exogenous intake of AGEs is also of great importance, which specifically involves low-fat and low-sugar diets or smoking cessation. The combination of exercise and dietary adjustments may synergistically reduce AGEs accumulation, offering a new perspective for the development of more efficient combined intervention therapies. However, precise and customized protocols for populations with different health statuses, genders, ages, nutritional conditions, and exercise backgrounds still require further investigation. Clinical monitoring of AGEs levels (e.g., via skin autofluorescence detection) may serve as a predictive or evaluative biomarker for the progression of sarcopenia. Nevertheless, due to the differences in AGEs metabolism among the skin, blood, and skeletal muscle, as well as uncertainties regarding the relationship between AGEs quantification and the pathological stages of sarcopenia, additional clinical studies are needed to verify the accuracy of AGEs as a potential biomarker for sarcopenia. Furthermore, targeting the AGEs-RAGE signaling axis can mitigate the pathogenic effects of AGEs on skeletal muscle. The development of corresponding drugs may thus provide a novel therapeutic direction for patients who are unable to exercise. In conclusion, promoting lifelong moderate exercise and “AGEs-aware” dietary habits helps reduce the global burden of age-related or metabolism-related muscle atrophy, which aligns with the long-term goal of extending healthy lifespan.
